# Considerations for biobanking of nonhuman genome data connected to Indigenous Peoples and lands

**DOI:** 10.1111/cobi.70301

**Published:** 2026-05-06

**Authors:** Alida de Flamingh, Nathan Alexander, Joshua D. Diaz, Alyssa C. Bader, Ripan S. Malhi

**Affiliations:** ^1^ Center for Indigenous Science, Carl R. Woese Institute for Genomic Biology University of Illinois at Urbana‐Champaign Urbana Illinois USA; ^2^ Ecology and Evolutionary Biology University of Tennessee Knoxville Tennessee USA; ^3^ Illinois Natural History Survey, Prairie Research Institute University of Illinois at Urbana‐Champaign Urbana Illinois USA; ^4^ Department of Anthropology University of Illinois at Urbana‐Champaign Urbana Illinois USA; ^5^ Department of Anthropology Oregon State University Corvallis Oregon USA

**Keywords:** access and benefit sharing, culturally significant species, ethical research framework, Indigenous data sovereignty

Considerations and guidelines for biobanking (storing and disseminating) and management of genomic data related to Indigenous Peoples often focus on human and human‐associated biospecimen data data (Hudson et al. 2016; Beaton et al. 2017; Carroll et al. 2020). The platforms from which these considerations are disseminated seldom reach beyond life science and biomedical science to, for example, environmental science journals, such as *Conservation Biology*. Yet, many areas within environmental science rely on genomic data connected to Indigenous Peoples or Indigenous lands; thus, it is critical for the field to join these growing discussions and adapt or develop relevant best practices.

Environmental science researchers have called for more ethical engagement with species and biological systems of cultural importance (e.g., through the incorporation of Indigenous perspectives and relations with such species) (Goolmeer et al., 2022; Reyes‐García et al., 2023). Culturally significant species are plants and animals of material, spiritual, foodway, and relational importance that shape identity, knowledge systems, and traditional practices across generations (Coe & Gaoue, 2020; Gore‐Birch et al., 2020). One environmental science–focused example of ethical engagement with such species is the Strong Peoples—Strong Country framework, an Indigenous heritage monitoring system that links the health of the Great Barrier Reef and its catchments to the well‐being of traditional owners or groups who have ancestral, cultural, spiritual, and custodial connections to particular tracts of land and sea). Grounded in local context, community perspectives, and governance, it recognizes cultural, spiritual, and ecological knowledge and positions Indigenous leadership beyond tokenistic participation (Jarvis et al., 2019).

Inspired by this and other culturally grounded approaches, we propose expanding current research frameworks to support more ethical and community‐engaged genomic studies of culturally significant species. In particular, we advocate extending human‐focused genomic guidelines and considerations to include nonhuman organisms, such as plants, wildlife, insects, and other taxa. We focus on one way in which genome science can accomplish this, which is through recognizing and respecting Indigenous data sovereignty (IDS) and the impacts of data dissemination on nation and community livelihoods and ensuring access and benefit sharing (ABS) when such data are deposited into biobank repositories. IDS is the right of Indigenous Peoples to control data about them, including data from their lands, resources, cultures, or genetic materials (Carroll et al., 2020; Halmai et al., 2025). ABS ensures that Indigenous communities receive fair and equitable benefits when their genetic or biocultural resources or related data are used (Sanghera et al., 2016).

Genomic data connected to Indigenous Peoples and lands have been, and continue to be, pervasive in academic research. Studies routinely analyze DNA from human remains (First Rider et al., 2024; Severson et al., 2022) and culturally significant species, for example, horses (Taylor et al., 2023), bison (Zimmerman et al., 2025), and salmon (de Flamingh et al., 2018), central to Indigenous lifeways and culture. These practices require particular attention in settler colonial contexts, for example, the United States, Canada, Australia, and Aotearoa (New Zealand), where Indigenous relationships to place remain politically and culturally foundational and where genomic research intersects with ongoing struggles for sovereignty, land rights, and jurisdiction (TallBear, 2013a; Tuhiwai Smith 2012; Wolfe, 2006).

Genome science is fraught with the mistreatment of data associated with Indigenous and at‐risk communities (Abadie & Heaney, 2015; Drabiak‐Syed, 2010; Mello & Wolf, 2010; TallBear, 2013b). Genomic data have been and continue to be collected, used, and shared in ways that harm and alienate Indigenous and other marginalized communities, who are underrepresented in fields of scientific research. Frameworks have been developed for Indigenous‐centered approaches to human genome research that should be followed when working with living and ancestral Indigenous communities (Bardill et al., 2018; Claw et al., 2018; Fleskes et al., 2022). Broadly, these approaches recommend developing research partnerships with Indigenous nations and communities to center their goals and interests as drivers of the research, ensuring these community partners are actively engaged through each aspect of the research process, and upholding the data sovereignty and governance of Indigenous research partners (who may attribute equal need for careful consideration of genetic data from all organisms, whether human or nonhuman). Although contextualized within human genome data, these frameworks for research with and by Indigenous Peoples provide foundational principles directly relevant and applicable to genome data from nonhuman species of sociocultural importance. For example, a relational framework has been developed for microbiome research with Indigenous communities (Bader et al., 2023).

Recently, there have been calls for consideration of IDS as part of wildlife conservation genetics (Robbins et al., 2023) and global biodiversity conservation (Mc Cartney et al., 2022; Walter et al., 2021). Robbins et al. (2023) provide actionable recommendations that include sovereignty, autonomy, and institutionalization (i.e., development of formal institutions that foster ethical accountability) for wildlife biopreservation (i.e., biobanking). These recommendations aim to support Indigenous Peoples and local communities in exercising authority and autonomy over genetic resources and associated biological data, including decisions about how such materials are collected, preserved, accessed, and used, in ways that align with their own governance systems, cultural values, and sovereign rights. Recommendations for generating, analyzing, interpreting, and biobanking nonhuman genomic data echo the priorities for human genome data in that they focus on integrating CARE (collective benefit, authority to control, responsibility, and ethics) (Carroll et al., 2020) and FAIR (findable, accessible, interoperable, reusable) (Wilkinson et al., 2016) principles so that data will remain under Indigenous control, serve Indigenous purposes, and reflect Indigenous ontologies and epistemologies (Robbins et al., 2023). These recommendations and principles stem from a multifaceted plea to counteract and prevent biopiracy, in which scientists and commercial entities extract and exploit Indigenous knowledge of biological materials for commercial or scholarly gain without authorization, acknowledgment, or remuneration of Indigenous Peoples for their contribution (Robbins et al., 2023; Robinson, 2014).

To understand the call and urgent necessity for implementing these principles, scientists first need to consider how nonhuman genomic data have and can affect Indigenous and other involved communities. Biobanks can aid in the preservation of nonhuman genetic diversity and are foundational to the stewardship not only of rare populations and endangered species in captivity and in the wild (Wildt, 2000), but also of economically important nonhuman species (Breithoff & Harrison, 2020). In a nonhuman animal context, examples exist in which biological data are associated with positive economic outcomes and benefits, including genetic data, that directly affect Indigenous communities (Matson et al., 2021; Schott et al., 2020; Schroeder et al., 2020; Shamon et al., 2022). For example, the reintroduction of bison (*Bison bison*) to Indigenous lands has generated substantial economic benefits alongside cultural and ecological gains (Shamon et al., 2022). The precipitous decline of bison resulted from mass commercial hunting, colonial expansion, and government policies aimed at undermining nations of Indigenous Peoples. Within a few decades in the 19th century, tens of millions of bison were reduced to a few hundred individuals (Feir et al., 2024; Isenberg, 2000).

Because many Indigenous nations’ cultures, economies, and foodways depended on these herds for millennia, their near extermination disrupted traditional lifeways, and bison restoration should include reparations and recognition of historical injustice. Initiatives such as the Wolakota Buffalo Range (Rosebud Sioux Tribe) and the Bison Range (Confederated Salish and Kootenai Tribes) have created jobs in habitat management, animal care, and ecotourism and generated revenue from visitors and sustainable bison‐related products. These projects demonstrate how ecological restoration can directly advance the economic goals of Indigenous communities by, for example, enhancing land productivity through managed grazing and habitat restoration, diversifying local income streams, and building community capacity for long‐term economic independence (Shamon et al., 2022).

In the case of bison, genetic data and genetic management have been integral to species restoration efforts, ensuring the long‐term viability and ecological function of reintroduced populations (Scheideman et al., 2025). Despite historical bottlenecks, bison populations maintain substantial genetic diversity, which is crucial for their adaptability and survival in changing environments (Scheideman et al., 2025). The use of whole‐genome sequencing has identified instances of hybridization between bison and domestic cattle, underscoring the importance of genetic monitoring to preserve the genetic integrity of bison herds (Stroupe et al., 2022). Furthermore, the implementation of translocation strategies, informed by genetic assessments, enhances gene flow among isolated bison populations, thereby bolstering their genetic health and resilience (Scheideman et al., 2025).

The direct link between the economic, cultural, and ecological gains discussed by Shamon et al. (2022) and the utility of genetic data and the associated insights stored and disseminated through biobanks highlights why it is critical for environmental science to engage with discussions of ethical biobanking practices. Another example, which importantly extends considerations beyond economic goals, can be found in a case study by Collier‐Robinson et al. (2019), who embedded Indigenous principles in genomic research on culturally significant taonga (sacred) species, the *kōwaro* (Canterbury mudfish [*Neochanna burrowsius*]) and *kēkēwai* (freshwater crayfish [*Paranephrops* spp.]), by developing a research program with the Ngāi Tūāhuriri subtribe that integrates Māori knowledge with genomic technologies and ecological data for species and biodiversity conservation (also see Rayne et al. [2022] and Hutchins et al. [2025]). These and other examples (Matson et al., 2021; Schott et al., 2020; Schroeder et al., 2020) highlight the importance of considering ABS (Carroll et al., 2024) and IDS (Colella et al., 2023) in the context of Bioanking (the storage and dissemination of such data).

IDS and ABS can also be prioritized through Indigenous biobanks under Indigenous stewardship, such as the Native BioData Consortium (https://nativebio.org) in the United States and the Clinical Research Laboratory and Biobank overseen by the Alliance National Aboriginal Working Group (NAWG) in Canada (https://cahhm.mcmaster.ca/first‐nations‐cohort‐indigenous‐BioBanking). Unlike other genomic databases that prioritize broad data sharing, Indigenous biobanks require community‐level consent and culturally appropriate governance, and emphasize benefit sharing, capacity building, and respect for Indigenous values (Caron et al., 2020).

Projects such as the Silent Genomes Project in Canada and the Aotearoa Variome in New Zealand show how these frameworks enable equitable research participation, contextualized consent and oversight, and improved health outcomes for Indigenous Peoples (Caron et al., 2020; Halmai et al., 2025). Large‐scale biobanking projects, such as the Earth Biogenome Project, should also continue open discussions about IDS and ABS (https://www.earthbiogenome.org/data‐sharing‐management‐best‐practices). In addition, biocultural labels, as described by Anderson and Hudson (2020), support IDS and ABS by embedding Indigenous provenance and cultural responsibilities in genomic data, including digital sequence information. These labels make visible “the provenance and ethics of collections; outline community expectations and consents about appropriate use; connect data to people and environments; and enhance Indigenous control over Indigenous data” (Anderson & Hudson, 2020). Finally, considerations for biobanking genomic data can also draw from existing frameworks of ethical standards that include meeting the relevant institutional, organizational, or community ethical requirements, such as approval from an institutional review board, an Institutional Animal Care and Use Committee (Institute of Laboratory Animal Resources 1986; U.S. Department of Health and Human Services 2018), or the relevant community‐specific research review council or board.

Concerns about biobanking and genomic research, whether involving human or nonhuman samples, have been frequently and consistently raised by Indigenous communities. As Garrison et al. (2019), Fox (2020), and Hudson et al. (2023) show, Indigenous scholars and communities themselves are also calling for research practices that respect cultural values, uphold sovereignty, and ensure meaningful benefit sharing. This is particularly significant given the findings of a global study by Reyes‐García et al. (2023), which showed that culturally important species are most prevalent among Indigenous Peoples. Results of this study also underscore calls to integrate biocultural approaches into conservation science and the lack of effective mechanisms for doing so. We argue that incorporating more ethical research principles, such as IDS and ABS, when biobanking genomic data of culturally important species is one way in which environmental science researchers can help achieve this. As inclusive genome research is pursued that benefits a greater proportion of our global society, existing human‐focused biobanking guidelines need to extend to nonhuman systems to safeguard IDS and ABS actively and effectively. The guidelines proposed by Robbins et al. (2023) and others (e.g., Carroll et al., 2020; Claw et al., 2018; Fleskes et al., 2022; Garrison et al., 2019; Hudson et al., 2016; Wilkinson et al., 2016) provide a valuable foundation from which to deepen this discussion. We acknowledge that this discussion does not cover all potential considerations when biobanking nonhuman data; however, our goal was to present researchers with resources to consult to further understand inclusive and ethical considerations of biobanks and other genome repositories and to create a space for initiating dialogue about prioritizing sociocultural importance, IDS, and equity through ABS when depositing genome data into biobank repositories.
